# Exploring the Experiences of Moderators From an Asynchronous Online Dementia Support Forum: Qualitative Interview Study

**DOI:** 10.2196/94218

**Published:** 2026-07-07

**Authors:** Catherine V Talbot, Neil S Coulson

**Affiliations:** 1 Bournemouth University, Poole, England, United Kingdom; 2 Clinical Sciences Building, University of Nottingham, Nottingham, England, NG3 3AT, United Kingdom, 44 0115 951 5151

**Keywords:** dementia, online peer support, moderation, online communities, volunteer support, caregiving

## Abstract

**Background:**

Asynchronous online forums provide flexible, accessible peer support for many people living with dementia and carers. Moderators are central to the functioning of these communities, yet little is known about their experiences.

**Objective:**

This study explored the experiences of individuals moderating an online dementia forum, including their motivations, perceived benefits, challenges, and suggestions for improvements.

**Methods:**

Moderators from a UK-based online dementia support forum were recruited using purposive sampling via forum administrators. Between January and March 2025, 5 moderators, all with dementia care experience, participated in remote semistructured interviews. Interview topics included pathways into moderation, perceptions of the moderator role, experiences of supporting forum members, challenges encountered, perceived personal benefits, and views on the future development of online support communities. Interviews were transcribed verbatim and analyzed using reflexive thematic analysis.

**Results:**

Four themes were produced: (1) “from support seeker to support provider”: moderators primarily identified as community members rather than authority figures, following a trajectory from receiving support as a carer to actively facilitating community support; (2) “understanding through shared experience”: lived experience of dementia was seen as essential for empathy, credibility, and sensitive responses, though sometimes prompted strong emotional reactions; (3) “giving back and gaining in return”: moderation offered purpose, structure, and social connection, particularly postretirement and following transition out of caring; (4) “balancing growth with community preservation”: forum expansion increased workload, spam management demands, and safeguarding responsibilities, and moderators were cautious about social media–style features and artificial intelligence–generated content undermining the effectiveness of support exchanges.

**Conclusions:**

Moderators play a crucial, value-driven role in sustaining dementia support forums, extending beyond administrative duties. The findings suggest that moderators occupy a distinctive position as both recipients and providers of peer support, drawing on experiential expertise to maintain trust and community cohesion. Forum growth and technological innovations present opportunities and challenges, highlighting the need to balance scalability with authenticity in online support communities.

## Introduction

Asynchronous discussion forums have become an increasingly prominent form of online peer support, providing flexibility for users to engage with similar others at their own pace, convenience, and often anonymously [1,2]. Unlike synchronous communication platforms, which require real-time participation, asynchronous forums allow users to post messages and respond in their own time, removing barriers for those managing complex health conditions, caregiving responsibilities, or differing time zones [3]. These platforms can help create a sense of community and belonging, enabling users to find validation, exchange social support, and reduce feelings of isolation [4,5]. Participation in asynchronous forums can also enhance feelings of safety and autonomy, as users have greater control over what and how much they disclose [6,7]. Furthermore, the written, persistent nature of forum content creates a searchable, evolving repository of experiential knowledge, which can benefit both active participants and passive readers [8].

In recent years, there has been growing recognition of the value of online peer support for people affected by dementia, including those living with the diagnosis and carers [9,10]. With an estimated 55 million people living with dementia worldwide [11], there is a pressing need for scalable approaches to support those affected in managing the cognitive, emotional, and social challenges associated with the diagnosis [12-14]. Online peer support appears to fill gaps in current offline dementia service provision, offering users opportunities to connect across geographical distances, engage flexibly, and foster a sense of belonging without the need for travel [10,15]. Asynchronous platforms also allow users to reach a wide audience facing similar challenges, who may otherwise be difficult to reach offline, and provide a degree of anonymity that can help overcome the stigma associated with dementia [16]. Analyses of asynchronous online support forums indicate that they enable people with dementia and carers to disclose personal experiences and access timely, appropriate, and reciprocal peer support [17,18].

Moderators are central to the effective functioning of asynchronous online peer support forums, helping to ensure that these spaces remain respectful, inclusive, and emotionally safe for users. They typically fulfill multiple responsibilities, including welcoming new members, guiding discussions, managing interpersonal conflict, addressing inappropriate or harmful content, and modeling supportive communication behaviors [19,20]. While most research has focused on moderator activities or message content, some studies have examined their lived experiences, particularly in mental health contexts. These studies show that moderators are often motivated by a desire to help others and find the role meaningful, but also emotionally demanding, with effective moderation requiring empathy, sensitivity, and care [21,22]. However, to our knowledge, the experiences of moderators within dementia-specific forums have not yet been explored. This is important given the nature of discussions within dementia forums, which can involve emotionally complex topics such as diagnosis, cognitive decline, identity changes, and caregiving, with contributions from users with a variety of cognitive impairments and caring experiences [18]. As many people affected by dementia face barriers to accessing offline support [23], moderators may also play a particularly important role in maintaining safe and supportive peer environments. Understanding their experiences is therefore critical to supporting the sustainability and effectiveness of dementia-related online communities. Therefore, in this interview study, we aimed to explore the experiences of moderators of an online dementia support forum, including their motivations, perceived benefits, challenges, and suggestions for improving moderation processes and forum infrastructure.

## Methods

### Study Design

A qualitative design, using semistructured individual interviews, was selected to explore the subjective experience of forum moderators.

### Participants and Recruitment

Participants were recruited from a pool of 11 moderators of a widely used online forum, operated by the leading dementia charity in the United Kingdom. Although participation is not formally restricted to residents of the United Kingdom, the asynchronous forum is primarily oriented to the United Kingdom, and discussions often reference health care, social care, and legal systems within this country. Within the forum, there are 2 roles relevant to the research aim: “hosts,” who provide additional support to the forum and its members, and “moderators,” who are responsible for creating a welcoming and safe environment, offering support, and enforcing forum rules where necessary. For the purposes of this study, both roles were classified as moderators because they share core responsibilities related to peer support and community engagement. To initiate recruitment, the online forum manager was contacted to first obtain permission for the study. Following this, a summary of the research project and link to the online participant information sheet and consent form were sent to the gatekeeper, who then distributed these materials to moderators. As recruitment relied on moderators voluntarily responding to the invitation, participation in our study was therefore based on self-selection. Therefore, our sample may overrepresent moderators who were more actively engaged with the forum, had stronger views on the forum, or were more willing to discuss their experiences within the research context. A total of 5 participants were recruited from the pool of moderators, with an average age of 72 (SD 6.91) years. All participants had experience of caring for a family member with dementia. To protect confidentiality, no additional demographic information is reported due to the small and identifiable nature of the moderator group.

### Data Collection

After providing informed consent, participants were invited to take part in a remote interview via Zoom with the first author. Interviews lasted up to 60 minutes and were conducted between January and February 2025. A semistructured interview schedule (Multimedia Appendix 1), developed in collaboration with the gatekeeper, was used to guide discussions. The questions were also informed by the findings of recent research examining online support exchanges within a dementia support forum [17,18]. At the start of each interview, participants were reminded that the research was being conducted independently of their organization and that their responses would remain anonymous. Participants were asked about their role and responsibilities, motivations for becoming a moderator, the perceived benefits of the forum for users and themselves, any challenges associated with the role, and any changes that could improve their experience. Interviews were recorded using Zoom’s built-in recording feature and auto transcription. The first author then reviewed each transcribed interview to ensure accuracy, with only minor amendments required.

### Data Analysis

Interview transcripts were analyzed using reflexive thematic analysis to identify patterns of meaning across the data [24]. Consistent with a reflexive thematic approach, we acknowledge that themes were actively generated through our engagement with the data rather than being discovered as objective entities [25]. The first author has extensive experience conducting research with people living with dementia and carers, while both authors have personal experience supporting family members affected by dementia. These experiences contributed to a belief in the value of peer support and an appreciation of the challenges associated with dementia and caregiving. In addition, both authors have used online forums as sources of information, support, and community, which may have sensitized us to the potential benefits of online peer support environments. Throughout the analysis, we engaged in ongoing discussion and reflection regarding how our experiences, assumptions, and expectations might shape the interpretation of the data. For example, we were mindful of the possibility that our positive views of peer support could lead us to overemphasize beneficial aspects of moderation, and therefore paid particular attention to accounts describing challenges, tensions, and negative experiences. Reflexive discussions between authors were used throughout coding, theme development, and interpretation to critically examine emerging understandings and consider alternative interpretations of the data.

Both authors began by reading all interview transcripts to support in-depth familiarization, noting initial observations which were later discussed. The authors then independently coded each transcript before meeting to discuss their interpretations. These discussions focused on exploring different readings of the data and refining coding rather than seeking coding consensus. Where the authors interpreted the data differently, these perspectives were discussed reflexively to further develop the analysis. Codes were visually mapped into preliminary themes by the first author using Miro [26], an online platform with a virtual whiteboard feature. This map was shared with the second author, and themes were refined through collaborative discussion. This iterative process resulted in a more focused visual map in which core concepts and definitions were clarified for each theme. Themes were reviewed against the original dataset to ensure coherence. Following further refinement, themes were named, and the analysis was written up. An audit trail was maintained throughout the analysis, documenting theme development.

### Ethical Considerations

Ethical approval was received from the University of Bournemouth (ID: 61149). All participants provided written informed consent and received a GBP £15 (GBP £1=US $1.31 as of June 25, 2026) voucher as compensation for participating in the study. To promote participant privacy, the names of the specific forum and organization are not provided.

## Results

### Summary of Themes

Our thematic analysis produced four themes: (1) “We are first and foremost members”: from support seeker to support provider; (2) not just IT support: understanding through shared experience; (3) giving back and gaining in return: finding value through moderation; and (4) balancing growth with community preservation (Table 1).

**Table 1.** Overview of themes.

Theme name	Definition
We are first and foremost members: from support seeker to support provider	Moderators primarily identified as community members rather than authority figures, with their role evolving organically from their own experiences of seeking support.
Not just IT support: understanding through shared experience	Moderators understood their role as providing emotionally attuned, experience-based support rather than purely technical or administrative assistance.
Giving back and gaining in return: finding value through moderation	Moderators were motivated by a desire to give back to a community that had previously supported them, while also deriving personal benefits, including a sense of purpose and continued social connection.
Balancing growth with community preservation	Moderators navigated tensions arising from the forum expansion, technological innovation, and maintaining the community’s supportive ethos.

### We Are First and Foremost Members: From Support Seeker to Support Provider

Participants reflected on their experiences of initially engaging with the forum as community members before taking on a moderator role, describing a trajectory that began as support-seekers while navigating the complex and often isolating experience of caring for someone with dementia. As community members, participants initially joined the forum feeling overwhelmed, lost, and unsure of where to seek help. The forum was described as a “lifeline” during this early phase of engagement, offering practical advice, emotional support, and connection with others who understood their experiences without judgment.

I joined the forum to start off with, obviously for help with my mom, because I didn’t know where to turn.

[P1]

I joined shortly after my husband was diagnosed and I had lots of questions. I was lost. I didn’t know where to turn and the members just made me feel, yeah, we’re here, we can help you.

[P3]

The forum was consistently described as a safe and supportive environment where community members can express themselves freely, ask questions, share concerns, vent frustrations, or simply “get things off their chest” (P1). Anonymity was seen as particularly important in enabling members to speak openly and honestly about their experiences. The sense of safety was grounded in the shared experience of dementia, fostering acceptance, empathy, and mutual understanding.

The connection with other members, the understanding. People who understood what you were going through. No judgments made, you know … If you had a question, a concern, or just wanted to let off steam, you could go on to the forum, and you would know that you would be understood, and members would come on with suggestions, advice, stuff you didn’t have to follow, but it made you feel less alone.

[P2]

Over time, participants transitioned from receiving support to actively contributing to the forum. As they grew more confident and familiar with the space, many drew on their own experiences to offer meaningful support to others in similar situations. For some, this ongoing engagement led to an invitation to become a moderator, whereas one participant responded independently to a general call for volunteers.

One of the things that I was told when I was first approached was that they liked the way that I responded to people. That they thought I had some empathy towards them. That I understood the issues that carers do have.

[P3]

While moderators performed essential administrative tasks, such as reporting posts and enforcing guidelines, they played a much broader role in sustaining the forum by facilitating support exchanges and maintaining a strong sense of community. They saw themselves as “first and foremost members” (P3), viewing moderation as an extension of forum participation rather than a separate authority. They worked to ensure every post received a response; no question was left unanswered; and all members, particularly newcomers, felt welcome and heard. For them, the moderator role was defined by presence and engagement rather than authority.

It’s important that new members get a reply very quickly … Because I wouldn’t want them to feel they were being ignored, or there’s nobody there. They need to know that there’s somebody there.

[P4]

It’s just sort of being able to really make somebody feel welcome on the forum, and being, as you know, of help, of assistance to them. It may be a crisis, or it may just be a simple question that someone’s put.

[P5]

Notably, most moderators were no longer active carers, yet they remained strongly engaged with the forum and committed to sustaining its supportive ethos. Becoming a moderator was seen as a natural continuation of their journey with dementia, grounded in a commitment to preserving the community that had been so valuable to them as members and to sharing their knowledge with those now facing similar challenges.

### Not Just IT Support: Understanding Through Shared Experience

Participants identified several qualities as essential for a successful moderator, including being nonjudgmental, calm, collaborative, and a good listener. However, the most consistently valued trait was lived experience of dementia, with all moderators having caring experience. This experiential knowledge enabled them to connect with members on a personal level, anticipate needs, respond sensitively, and create an environment where users felt heard and understood. Moderation, therefore, extended beyond administrative or technical tasks; it was fundamentally grounded in shared experience and understanding.

I think it’s very beneficial to have that experience. Because you can understand why people are putting what they put. You know, a lot of the time I’ve been there, or all the moderators have been in those sorts of situations … Rather than it just being an IT experience of “this doesn't fit the rules.”

[P1]

A key benefit of lived experience was its role in facilitating empathy within support interactions. By genuinely understanding what members were going through, moderators could respond authentically, drawing on their own caring experiences to provide informational and emotional support. While most moderators were former carers who could still draw upon past experiences to connect meaningfully, one current carer highlighted the added value of relating directly to members’ present-day challenges, given recent changes to formal dementia support provision.

So, say if you were a carer 10, 15 years ago. Things were a lot different than they are now. There was a lot more help available 15-20 years ago than there is now, so I probably understand that. And yes, the other members do understand it as well. But when they were carers, they had more options than perhaps the carer now does.

[P3]

Empathy derived from lived experience was seen as central to moderators’ credibility within the community. Participants expressed concerns that moderators without firsthand experience might struggle to connect with members, lacking the authenticity and depth of understanding required. While professional or academic knowledge was valued, it was considered distinct from personal experience, and participants worried this gap could diminish the quality of support exchanges. Lived experience was, therefore, regarded as essential for establishing empathy, trust, legitimacy, and effective support exchanges.

I think they’d have more difficulty with the members of the forum, because they wouldn’t come across as having any experience. We post as members as well as moderators. So, I don’t think they’d have a lot of credibility. And I would worry that their posts would become more academic sounding, and therefore almost sound as if there was no empathy.

[P2]

Strong communication skills were also highlighted as essential for responding sensitively and avoiding language that might seem blunt or dismissive. This emphasis suggests the presence of a shared community language, shaped by mutual understanding and a commitment to supportive interactions. Moderators were attuned to this code, adapting their responses to align with the tone and expectations of the community.

Somebody who is sensitive and tactful. There are ways of putting sentences together where, rather than being too blunt about … if you’re giving advice, or a suggestion, it’s got to come across as that, and not as prescriptive as you must, or you should do this or that. Sometimes, by just using the phrase “in my experience,” or “what I found was.” But I think they’re the main sort of experience: having empathy, being able to put a post together which is sensitive and which, you know, shows compassion.

[P5]

Despite its benefits, lived experience sometimes presented challenges, particularly when posts resonated too closely with moderators’ own caring experiences. These moments sometimes triggered strong emotional reactions, prompting participants to step back to protect their well-being. The availability of formal mental health support (ie, a dedicated counselor) was highly valued and provided an essential resource for sustaining moderators’ well-being. These accounts highlight the emotional labor involved in moderation and the importance of formal support structures.

There are times where I’ve had to walk away from the forum, from the computer for the rest of the evening or morning, or whatever because it can be, sometimes it could be very close to home. For instance, just a week, 10 days ago, somebody posted about their mum … And it so resonated, it could have been my mum in the early days, it really could. And so, to really reply, saying you know how it resonated with me, and why, and try to offer some suggestions or advice of what this person may do and how to help. And again, after that I did have to sort of walk away from the forum for a little bit.

[P5]

Although the forum supports people living with dementia and carers, none of the moderators had a diagnosis themselves. Some participants saw this as a potential area for development, noting that people with early-stage dementia could, with appropriate support, take on a moderator role and their lived experience could further strengthen empathy within support exchanges.

### Giving Back and Gaining in Return: Finding Value Through Moderation

Moderators commonly described their involvement as a way to give back to the community that had supported them during their own experiences with dementia. Their transition from forum user to moderator was often driven by gratitude and a desire to help others facing similar challenges. Many also reported that the role provided a renewed sense of purpose, particularly following retirement or bereavement, and was experienced as fulfilling, meaningful, and emotionally rewarding. Positive feedback from forum members reinforced the value of their contributions and sustained their motivation to continue.

I’d retired when I was 58 and then obviously ended up looking after my mum, looking after my dad. And you know, things like that. And it was … I didn’t have a purpose. I didn’t know what my purpose was, if that makes sense. And this just came up as an opportunity. The fact that I was asked to do it made me think, yeah, somebody recognised something.

[P1]

The feedback I get back. I get very, very good feedback, you know. People will say, Oh, you’ve helped, or thanks for that link.

[P4]

Moderating was often described as a daily activity that brought structure, particularly for those who were retired or housebound. Participants also described how their role helped them to continue learning, encountering new information and experiences through the forum.

I do 2 hours in the morning when I get up. I get up in the morning and have my tea and coffee, and spend a couple of hours on the Forum, and then I switch the television off at 10 o'clock, and I spend a couple of hours in the evening, and then through the day I dip in and out on my phone … So it is a lot, but it’s at least 4 hours a day, every day.

[P4]

You learn something new all the time. Yeah, something will come up in the forum. You go right, I’ve never come across that for how do you, you know, what advice can we give there?

[P3]

Participants spoke fondly of the relationships they had created, both with fellow moderators and forum users. It was evident that these connections provided emotional support, social engagement, and a shared sense of purpose. Several described how the forum was a space where they felt known, trusted, and appreciated.

By being on the forum so much, people get to know me, and it’s the same with other moderators. You know, we’re on a lot, and they know us, and so they feel that they can offload a little bit more.

[P4]

### Balancing Growth with Community Preservation

Moderators reflected on the forum’s substantial growth in recent years, which they largely saw as positive, signaling its relevance and importance as a supportive resource for people affected by dementia. However, expansion also brought challenges. Participants reported that the moderation team had not grown alongside membership, increasing pressure on existing volunteers. One moderator explained that what had begun as a few hours each morning now involved “going on it far more” (P1), spending 4 to 5 hours a day to keep up with demand. Ensuring timely responses to new members, maintaining a safe space, and upholding community standards had become increasingly difficult without additional resources.

There aren’t as many of us now. There used to be, I think, about eight moderators, and over time that’s gone down now. We just have the five. But we have more members.

[P3]

Alongside these demands, moderators consistently identified spam as a growing problem. While obvious posts promoting drugs or external links were easy to detect, their frequency had increased substantially with the forum’s popularity. Some spammers used subtler tactics, such as posting innocuous comments across multiple threads before inserting links. Managing spam, though routine, was time-consuming and frustrating, detracting from the moderators’ ability to provide meaningful support to members. The increase in spam highlighted the need for more robust systems or additional moderators to maintain the forum’s integrity amid rapid growth.

The responsibilities now are far different to, I mean, we hardly had any spammers in those days, so that responsibility is big now. We’ve just sorted one out now, they don't show up anything on Stop Forum Spam. They managed to avoid that! And yet they post 10 posts in quick succession, giving one-word answers to lots of different, and then you get the link. So, it means that we have to be really, really aware.

[P4]

Moderators highlighted the importance of appropriate safeguarding support, given the vulnerability of many forum users. Several participants described situations where new members appeared distressed or disclosed concerning information, prompting referrals to staff for further action. Others noted that while they could escalate concerns, they relied on staff to follow up, and not all felt confident navigating complex safeguarding issues alone. Regular training sessions, such as those on safeguarding procedures, were valued, but participants felt that clearer guidance and more consistent support from staff would enhance their ability to respond appropriately and maintain a safe forum environment.

Some of the threats of suicide were made outside staff hours. So that is extremely difficult to deal with because you feel as if you’re on your own, and you've nowhere to turn to.

[P3]

Moderators described managing challenging members as one of the more emotionally demanding aspects of their role. These members might post unsupportive comments in response to distressing situations or regularly test the boundaries of acceptable behavior. Moderators often relied on other users to report such incidents, but these reports were not always made, meaning problematic behavior could go unaddressed and some members might feel unwelcome and disengage from the forum. While moderators could escalate incidents, decisions about further action often rested with staff, which sometimes led to differences in judgment. Although staff support was appreciated, moderators suggested that clearer communication and shared decision-making would help address member concerns more swiftly and reinforce their own confidence in managing challenging interactions.

Challenging members can be extremely challenging … and she’s been really, really rude to people. So, these are the big challenges that I’ve not met before in all the years that I’ve been on the Forum.

[P4]

How do we know if members are upset or not? Some members might be too nervous, too anxious and not report, and just leave the Forum. We don’t know that, there’s no way of knowing that.

[P2]

Moderators reflected on how technological developments were shaping expectations for online forums, with a perceived increasing pressure to modernize platforms while retaining their central core supportive ethos. Participants shared concerns about the inclusion of design features typically seen on social media, such as “badges,” “likes,” and “reactions.” Several moderators considered these features inappropriate in the context of a dementia peer support forum, where members may post during moments of distress or in times of crisis. Such features could potentially encourage superficial responses and detract from the forum’s ethos of empathy and understanding.

There is talk of introducing sort of emojis where someone will write something, you know someone writes a post, or whatever, and rather than somebody responding with, you know, words, somebody can add a tick, you know, or thumbs up, or a heart, or whatever. I wouldn’t be happy with that, because it will, in my view, it’ll devalue the effect of the Forum. The effect of the Forum should be that people are writing. There’s empathy, there’s people.

[P5]

Some moderators raised concerns about the potential use of artificial intelligence (AI) within the forum. Participants worried that AI-generated responses would lack the authenticity and empathy central to peer support, potentially diluting the value of this space. These concerns reflected broader anxieties about preserving the forum’s ethos of genuine, experience-based connection amid technological change.

There needs to urgently be a policy on the use of AI in the forum … it doesn’t show any person, there are no personal feelings shown. There is no empathy in their replies to people, and that is, I think, what would be lacking if AI was allowed to creep in too much.

[P5]

## Discussion

### Principal Findings

This interview study aimed to explore the experiences of moderators of an online dementia forum. Our findings indicate that the moderator role was viewed as an extension of forum membership, rather than a distinct or authoritative position, grounded in the shared lived experience of dementia with other members. Moderators typically transitioned from support-seekers during their time caring for someone with dementia to support providers within the online community, drawing on experiential knowledge to offer empathetic, credible, and responsive peer support. While moderation provided benefits such as purpose, structure, and social connection, it also involved significant emotional labor and time commitment. Moderators also raised concerns about growing pressures associated with forum expansion and technological developments, including increased workload, spam management, safeguarding responsibilities, and tensions between technological innovation and preserving the forum’s supportive ethos. Our findings highlight that forum moderation is a relational, value-driven practice that is central to maintaining the safety, sustainability, and effectiveness of online dementia support spaces.

### Comparison With Prior Work

Moderators described a trajectory from initially using the online forum to seek support while caring for someone with dementia to becoming support providers within the community. Prior to taking on the moderator role, the forum was a vital source of support, offering practical advice, emotional support, and connection with others who truly understood their situation. This finding is consistent with research demonstrating the value of online forums for people affected by dementia, by facilitating timely peer support and opportunities for self-disclosure [17,18,27]. Over time, participants began to provide support to others within the community, and most took on the role following the death of the person they cared for, providing an extension of their personal journeys with dementia and a sustained sense of community belonging beyond the active caring period. Jameson et al [28] reported that carers often struggle with transitioning out of a carer role, experiencing a loss of identity and diminished social networks, yet seek opportunities to give back to others. Moderating online dementia communities may therefore provide the continuity of carer identity following the transition out of active caring, enabling continued meaningful connection to dementia communities while reshaping, rather than losing, that identity. Our findings extend current understandings of online support communities for dementia by illustrating how moderation can function as an active, identity-maintaining practice following the end of caregiving.

Moderators consistently emphasized that effective moderation relied heavily on having lived experience of dementia, mirroring peer support research identifying experiential knowledge as a distinct and valued form of expertise within health-related communities [29]. Participants in our study described how experiential knowledge shaped *what* support was provided and *how* it was communicated, with careful attention to tone, language, and emotional sensitivity. Empathy has been identified as a key mechanism in online peer support, helping others to feel understood, validated, and connected [30,31], and expressions of empathic concern have been found to predict more positive emotional responses among support seekers [32]. Our findings extend previous work on online dementia support communities, by demonstrating that experiential knowledge is not only an important resource among members [18] but also within moderation practices, where it shapes the provision of emotionally attuned support. Moderators with lived experience may therefore be particularly well positioned to facilitate meaningful and emotionally attuned support. However, lived experience also introduced emotional challenges for moderators in our study, particularly when posts resonated with their own caring experiences. This is consistent with research on the emotional labor of online moderation, including the strain of repeated exposure to distressing content and the risk of secondary trauma and burnout [33,34]. Given the critical role moderators play in sustaining online peer support spaces, their ability to provide effective support depends on moderators being adequately supported themselves. This highlights the importance of formal support structures, such as supervision, peer support networks, counseling access, and clear role boundaries, to safeguard moderators’ well-being and enable them to continue their roles safely and effectively.

Moderators frequently described their involvement as a way of giving back to a community that had previously supported them, highlighting the reciprocal and cyclical nature of online peer support. This reflects patterns observed by Yang et al [35], who found that members often transition over time from seeking support to providing it, suggesting that moderation may represent a continuation or formalization of this helping role. In addition to altruistic motivations, moderators derived personal benefits, including a renewed sense of purpose and daily structure, which was particularly salient given that most were retired. This is consistent with evidence that retirement is associated with increased engagement in volunteering, as older adults seek to maintain valued social roles and meaningful activities [36]. Virtual volunteering, such as moderator roles, may be beneficial for some older adults as it reduces barriers related to mobility and travel that have previously been linked to lower retention in offline volunteering [37]. Consistent with Mukherjee [38], moderators in our study gained meaning, satisfaction, and social connectedness, suggesting that online moderation can facilitate fulfillment and social capital in later life. However, it is important to recognize that digital exclusion is a concern, as barriers related to digital access and literacy may limit participation for some older adults [38]. This highlights the importance of inclusive platform design and support to ensure that the benefits of virtual volunteering are accessible to a diverse range of older adults.

The rapid growth of the dementia forum in recent years highlights both its value and the increasing demand for dementia-specific peer support, particularly given gaps in offline provision [23]. However, expansion can create a resource imbalance, with growth in membership outpacing the capacity of volunteer moderation teams, meaning organizations must scale moderator recruitment alongside community growth. Moderators in our study also expressed resistance to forum modernization features such as “likes,” “reactions,” and “badges.” While features such as these have been recommended as mechanisms to incentivize participation and forum growth [39], moderators perceived them as incompatible with the forum’s ethos of empathy, authenticity, and mutual support, given that members often post during periods of distress. Our findings therefore contribute to understandings of peer support platform design by showing how engagement-driven features and scalable interaction may conflict with online communities, where authenticity, reciprocity, and experiential understanding form the foundation of meaningful support. Emerging concerns were also raised about the potential use of AI to generate posts, particularly around preserving authenticity and the integrity of peer interactions. Although AI has shown some promise in enhancing online health communities, including improving the quality and responsiveness of support [40,41], moderators in our study perceived AI-generated content as undermining the genuineness of lived experience that underpins peer support. These findings highlight the need for organizations to carefully evaluate technological developments to ensure that support forums prioritize care, trust, and community integrity over engagement-driven metrics or automated forms of interaction.

### Limitations

Several limitations should be recognized. Our recruitment strategy may have introduced potential sources of bias. Participation was voluntary, meaning that moderators who chose to take part may have been those with particularly positive experiences of the role, greater engagement with the forum, or stronger identification with the moderator community. Recruitment was facilitated through a forum gatekeeper, which may have further influenced who received, attended to, or felt comfortable responding to the invitation. Consequently, the perspectives of moderators who were less active, more critical of the forum, or less willing to engage in research may not be represented. These factors should be considered when interpreting the findings. Furthermore, although the sample was small in absolute terms (n=5), it represented almost half of the entire moderator population (5 of 11 moderators) within the forum. We drew on the concept of information power, which suggests that smaller samples may be sufficient when the study aim is focused, participants possess highly relevant experience, and the quality of dialogue is strong. The participants were all experienced moderators with direct knowledge of the phenomenon under investigation, providing rich and detailed accounts that enabled the development of meaningful themes. Nevertheless, we do not claim thematic saturation, consistent with reflexive thematic analysis, where the emphasis is placed on depth and interpretative richness rather than saturation as a marker of quality.

Additionally, participants were drawn from a single UK-based dementia forum, which may limit the transferability of the findings to other online support contexts, such as social media–based support groups [42,43], or to forums with different structures, cultures, or moderation models. Participants were older adults and predominantly former carers, and no moderators with a diagnosis of dementia were included, as people living with dementia were not part of the moderation team at the time of the study. Therefore, we cannot infer how moderators with a diagnosis of dementia might perceive or navigate the role. Finally, the absence of observational or longitudinal data means the study cannot capture how moderation practices and emotional impacts may change over time or in response to specific forum events. Future research could usefully address these limitations by including multiple forums, a broader range of moderator backgrounds, and complementary data sources such as longitudinal interviews or forum interaction data.

### Conclusions

This study provides one of the first in-depth explorations of moderators’ experiences within a dementia-specific online peer support forum. Our findings demonstrate that moderation in this context is a relational and value-driven practice grounded in lived experience, empathy, and community belonging. Moderators were integral to the community, with their role emerging organically from prior support-seeking and being sustained through a desire to give back. In doing so, they contributed to the practical functioning of the forum and the preservation of its supportive ethos. However, moderation also involved significant emotional labor, time commitment, and increasing responsibility at a time of growing membership and technological developments. These pressures illustrate the careful balance required to expand and innovate while maintaining a culture of care within online forums. As demand for scalable dementia support continues to grow, organizations must recognize moderators as central stakeholders in the sustainability of online communities.

## Supplementary material

10.2196/94218 Multimedia Appendix 1 Interview schedule.

## Abbreviations

AI: artificial intelligence
