# Supplementary material for: Exploring the Experiences of Moderators From an Asynchronous Online Dementia Support Forum: Qualitative Interview Study
Source: JMIR Form Res. 2026 Jul 7;10:e94218. doi: 10.2196/94218 (PMC13340571; doi:10.2196/94218)
Supplement: Multimedia Appendix 1 [file formative-v10-e94218-s001.docx]

**Interview questions**

**Demographics**

1. What is your age?
2. What is your gender?
3. What experience do you have with dementia?
4. How long have you been in your role as a moderator?

**Moderator role & Motivations**

1. Please can you start by telling me a bit about your role as a moderator?
2. Why did you first decide to volunteer as a moderator?
3. What keeps you motivated to continue moderating the online forum?
4. In your view, what makes a successful moderator in this kind of forum?
5. In your opinion, how important is it to have personal experience of dementia as a moderator?
6. Have you views of dementia changed as a result of moderating the forum?
7. What strategies do you use to maintain a support environment on the forum?

**Forum impacts**

1. What role do you think the forum plays in supporting people affected by dementia?
2. What personal benefits do you gain from moderating the forum?

**Challenges**

1. What are the biggest challenges you face when moderating the online forum?
2. Have you ever encountered any particularly difficult or sensitive situations on the forum?
3. Have you ever encountered misinformation on the forum?
4. Moderating a forum on this topic can be emotionally challenging. How do you manage this emotional aspect while moderating?

**Future improvements**

1. What could improve your experience as a moderator?
2. What features of the forum could be improved to make moderating easier?

**Closing questions**

1. What advice would you give to someone starting out as a moderator?
2. Finally, is there anything we haven’t covered today that you feel is important for me to know?
